# Reverse translation of phase I biomarker findings links the activity of angiotensin-(1–7) to repression of hypoxia inducible factor-1α in vascular sarcomas

**DOI:** 10.1186/1471-2407-12-404

**Published:** 2012-09-11

**Authors:** W Jeffrey Petty, Mebea Aklilu, Victor A Varela, James Lovato, Paul D Savage, Antonius A Miller

**Affiliations:** 1 Department of Medicine, Section on Hematology and Oncology, Wake Forest University Health Sciences, Medical Center Boulevard, Winston-Salem, NC, 27157, USA; 2 Department of Cancer Biology, Wake Forest University Health Sciences, Winston-Salem, USA; 3 Department of Biostatistical Sciences, Wake Forest University Health Sciences, Winston-Salem, USA; 4 Comprehensive Cancer Center of Wake Forest University, Wake Forest University School of Medicine, Winston-Salem, NC, USA

**Keywords:** Angiotensin-(1–7), Sarcoma, Placental growth factor

## Abstract

**Background:**

In a phase I study of angiotensin-(1–7) [Ang-(1–7)], clinical benefit was associated with reduction in plasma placental growth factor (PlGF) concentrations. The current study examines Ang-(1–7) induced changes in biomarkers according to cancer type and investigates mechanisms of action engaged *in vitro*.

**Methods:**

Plasma biomarkers were measured prior to Ang-(1–7) administration as well as 1, 2, 3, 4, and 6 hours after treatment. Tests for interaction were performed to determine the impact of cancer type on angiogenic hormone levels. If a positive interaction was detected, treatment-induced biomarker changes for individual cancer types were assessed. To investigate mechanisms of action, *in vitro* growth assays were performed using a murine endothelioma cell line (EOMA). PCR arrays were performed to identify and statistically validate genes that were altered by Ang-(1–7) treatment in these cells.

**Results:**

Tests for interaction controlled for dose cohort and clinical response indicated a significant impact of cancer type on post-treatment VEGF and PlGF levels. Following treatment, PlGF levels decreased over time in patients with sarcoma (P = .007). Treatment of EOMA cells with increasing doses of Ang-(1–7) led to significant growth suppression at doses as low as 100 nM. PCR arrays identified 18 genes that appeared to have altered expression after Ang-(1–7) treatment. Replicate analyses confirmed significant changes in 8 genes including reduction in PlGF (*P* = .04) and hypoxia inducible factor 1α (HIF-1α) expression (*P* < .001).

**Conclusions:**

Ang-(1–7) has clinical and pre-clinical activity for vascular sarcomas that is linked to reduced HIF-1α and PlGF expression.

## Background

Angiotensin-(1–7) [Ang-(1–7)] is a component of the renin-angiotensin system that has demonstrated anti-angiogenic activity in pre-clinical models [1-6]. Murine xenograft models have demonstrated that Ang-(1–7) exerts anti-angiogenic effects in a variety of cancer types [7,8]. While this drug has shown a broad spectrum of activity, the specific angiogenic hormone suppressed by Ang-(1–7) treatment varies according to the type of cancer being treated. In some cancer cell lines, vascular endothelial growth factor (VEGF) is suppressed by treatment while in others, placental growth factor (PlGF) is suppressed [7,8].

A phase I study was conducted to examine the tolerability and activity of Ang-(1–7) for the treatment of patients with advanced solid tumors refractory to standard therapy [9]. The tolerability was very good and clinical benefit was observed in four patients. Two of these patients had metastatic sarcomas. The group of patients with clinical benefit demonstrated a reduction in plasma levels of the PlGF over time following treatment. In contrast, PlGF levels did not significantly change over time in the group of patients without clinical benefit. Levels of VEGF did not significantly change over time in either group [9].

The signaling changes that trigger repression of PlGF or VEGF have not been well characterized. The clinical observation of PlGF suppression in patients with clinical benefit has compelled us to determine how this occurs [9]. To investigate this phenomenon, the current study was undertaken to reverse-translate the clinical findings back to a preclinical *in vitro* model. This was accomplished by identifying the cancer type most likely to achieve a biomarker response and then evaluating changes in angiogenic signaling following Ang-(1–7) treatment of a related cancer cell line.

Since Ang-(1–7) exerts its anti-angiogenic activity through regulation of different angiogenic hormones depending on cancer type in pre-clinical models, it is possible that clinical changes in plasma anti-angiogenic hormones could vary depending on the type of cancer treated [7,8]. Repression of VEGF has been identified in pre-clinical models, but reduction of plasma VEGF levels was not documented in analysis of the phase I study [9]. If changes in VEGF occurred only in certain cancer types, a significant effect on VEGF could have been underestimated by the analysis.

The current study tests whether the type of cancer being treated impacted the likelihood of achieving a biomarker response in the phase I study. Changes in VEGF, PlGF, and basic fibroblast growth factor (βFGF) were tested for interaction with cancer type. Based on these biomarker results, i*n vitro* studies were performed to confirm activity and evaluate mechanisms of action engaged in a vascular sarcoma cell line.

## Methods

### Study design and dose escalation

Patients with advanced solid tumors refractory to standard therapy were enrolled in the phase I study. The results of this study were previously reported [9]. Patients were ineligible if they were taking angiotensin converting anzyme (ACE) inhibitors or angiotensin II receptor blockers (ARBs).

Ang-(1–7) was administered by subcutaneous injection daily for five consecutive days on a 21 day cycle. Treatment was continued until disease progression or unacceptable toxicity. Planned dose cohorts were: 100 mcg/kg, 200 mcg/kg, 400 mcg/kg, 700 mcg/kg, and 1000 mcg/kg. A standard 3 + 3 dose-escalation strategy was utilized. Maximum tolerated dose was defined as the highest dose level at which no more than one of six patients experienced a dose-limiting toxicity. This study was approved by the Institutional Review Board of Wake Forest University and was registered with the National Cancer Institute PDQ Database and ClinicalTrials.gov as NCT00471562.

### Measurement of angiogenic hormone levels

Blood samples were drawn at time points immediately prior to treatment as well as 1, 2, 3, 4, and 6 hours after Ang-(1–7) administration. Samples were placed on ice and plasma was extracted within 30 minutes of collection. Plasma samples were stored at −80°C prior to performing biomarker analyses.

Hemolysis was assessed in all plasma samples and five samples from three patients were excluded from biomarker modeling due to hemolysis. Aliquots of plasma were assayed by a third party vendor (Pierce Biotechnology, Woburn, MA). Searchlight ELISA technology was used to prepare standard curves and quantify vascular endothelial growth factor (VEGF), placental growth factor (PlGF), and basic fibroblast growth factor (βFGF). Samples were blinded prior to shipping.

### Cell culture

Ang-(1–7) and Ang II peptides were purchased from Bachem (Basel, Switzerland), dissolved in sterile water, and stored at −20°C. EOMA cell lines were purchased from American Type Culture Collection (Manassas, VA) and passaged as recommended. These cells were cultured in Dulbecco’s minimal essential media (DMEM) containing 10% fetal bovine serum (FBS). These cells were cultured in a humidified incubator at 37°C with 5% CO2 and passaged every 3 to 5 days.

### Proliferation assays

Cellular proliferation was measured using the CellTiter 96 assay (Promega, Madison, WI). Assays were plated in sextuplicate replicates in 96 well plates at a density of 1,000 cells per well. Basal absorbance activity was measured immediately prior to treatment. Cells were then treated with Ang-(1–7), Ang II, or untreated as a control. Ang-(1–7) treatments were selected to represent a range of clinically achievable concentrations. Absorbance activity was measured after 72 hours in Ang-(1–7) treated, Ang II treated, and control cells. Proliferation was calculated by subtracting the baseline absorbance from absorbance measured following angiotensin or control treatments. Proliferation rates in treated cells were normalized to untreated control cells.

### PCR arrays

EOMA cells were independently treated with Ang-(1–7) at a concentration of 500 nM or untreated as a control. Cells were harvested after 24 hours and RNA was extracted using TriReagent (Invitrogen, Carlsbad, CA). RNA concentrations were measured, and RNA integrity was inspected by assessing 18S/28S ratios. Murine angiogenesis and murine endothelial cell biology PCR Arrays were purchased from SABiosciences (Frederick, MD) and real-time RT-PCR reactions were performed per the manufacturer’s protocol. A four-fold change in gene expression was used to identify genes that were regulated by Ang-(1–7) treatment.

Custom PCR Arrays (SABiosciences) were then designed to measure genes identified by these initial PCR Arrays as well as the VEGF and PlGF angiogenic hormones in quadruplicate replicate. Real-time PCR reactions were performed according to the manufacturer’s protocol. Outlying values defined as those greater than 2 standard deviations from the mean were excluded from statistical analyses. In cases where outlying values were observed, PCR Array experiments were repeated to confirm the results.

### Statistical methods

Biomarker levels over time were modeled after log-transformation, consideration of quadratic effects of time (after centering), and adjustment for plasma drug levels. A mixed effects model was used to tests for interaction of cancer type and biomarker effect. This potential interaction was controlled for presence or absence of clinical benefit and plasma drug levels. In cases where a significant interaction between biomarker and cancer type was observed, univariate regression analyses were performed separately for each cancer type. These regression analyses examined linear biomarker changes over time following drug administration. Two sample t-tests were performed to compare *in vitro* proliferation rates at each dose level of Ang-(1–7) and Ang II to untreated controls. Two sample t-tests were also performed to compare PCR Array measurements from Ang-(1–7) treated and untreated cells. All analyses were two-sided, and a P-value < 0.05 was considered statistically significant. P values were not corrected for multiple comparisons. Clinical biomarker analyses were performed using SAS v9.1.3 (SAS Institute, Cary, NC) and Stata v10.1 (StataCorp, College Station, TX).

## Results

### Patients

Eighteen patients were enrolled in the phase I study. The results of this clinical trial were previously reported [9]. Patient data were grouped according to cancer type and characteristics of these groups are displayed in Table 1. Cancers with only one case were grouped into the category of “other.” This included one patient with urachal carcinoma, one with anal carcinoma, one with head and neck cancer, and one with lung cancer. 

**Table 1:** Patient characteristics

**Characteristic N (%) or mean ± SD**	**Overall (N = 18)**	**Colorectal (N = 6)**	**Prostate (N = 3)**	**Sarcoma (N = 3)**	**Pancreatic (N = 2)**	**Other (N = 4)**
*Ang(1–7) Arm*						
100 mcg/kg	3 (17)	0	1 (33)	1 (33)	0	1 (25)
200 mcg/kg	3 (17)	1 (17)	0	1 (33)	1 (50)	0
400 mcg/kg	6 (33)	4 (73)	1 (33)	0	1 (50)	0
700 mcg/kg	6 (33)	1 (17)	1 (33)	1 (33)	0	3 (75)
*Sex*						
Female	6 (33)	1 (17)	0	2 (67)	0	3 (75)
Male	12 (67)	5 (73)	3 (100)	1 (33)	2 (100)	1 (25)
*Race*						
Black	3 (17)	1 (17)	0	1 (33)	0	1 (25)
White	15 (83)	5 (73)	3 (100)	2 (67)	2 (100)	3 (75)
*Age*	61.3 ± 12.0	60.6 ± 12.1	63.0 ± 13.1	59.9 ± 19.3	73.4 ± 0.5	55.7 ± 4.3

### Impact of hemolysis on plasma angiogenic hormone concentrations

Hemolyzed plasma samples were excluded from prior biomarker analyses in phase I study due to concerns that hemolysis could alter angiogenic hormone concentrations. To test this hypothesis, concentrations of VEGF, PlGF, and βFGF were compared in hemolyzed and non-hemolyzed blood samples. PlGF concentrations were significantly increased in hemolyzed blood samples (9.2 vs 21.6 ng/mL, *P* = .01). Concentrations of βFGF appeared to be increased but this effect did not reach statistical significance (6.5 vs 17.5 ng/mL, *P* = .07). Concentrations of VEGF were similar in hemolyzed and non-hemolyzed blood samples (91.8 vs 92.8 ng/mL).

### Biomarker analyses

A mixed effects model was established to examine whether cancer type impacted the changes in angiogenic biomarkers observed during the phase I study. Tests for interaction were performed using pre- and post-treatment biomarker measures according to cancer type after controlling for dose cohort, the presence or absence of clinical response, and plasma drug level. Pre-treatment levels of VEGF, PlGF and βFGF were not significantly different according to cancer type. However, post-treatment levels were significantly different according to the type of cancer being treated for VEGF (*P* = .001) and PlGF (*P* = .002).

**Figure 1:**
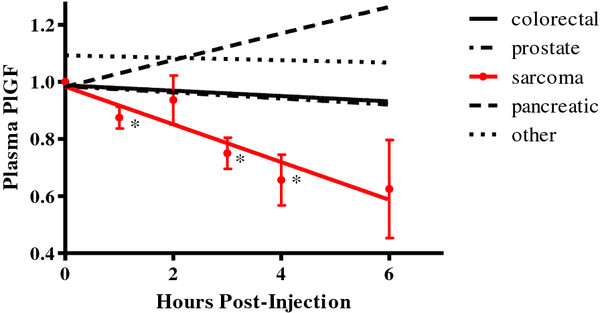
**Post-treatment PlGF changes according to cancer type on day 1 of treatment.** The lines shown represent trend lines for median normalized values. Red color indicates a significant trend (P < .05) based on the regression statistic. For cancer types with a significant trend, median values and standard deviation error bars are shown for each time point. * indicates a significant difference in biomarker value (P < .05) compared to the time 0 biomarker value based on a two-sample t-test.

**Figure 2:**
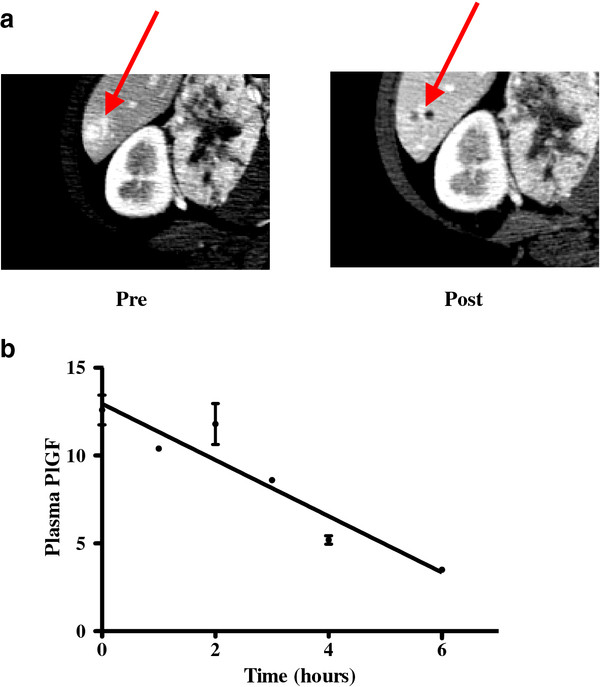
**(A) Radiographic changes in a patient with metastatic hemangiopericytoma after two cycles of treatment.** (**B**) Decreases in PlGF concentrations are shown for the same patient on day 1 of Ang-(1–7) treatment. Error bars represent the standard deviations of repeated ELISA analyses from a single plasma sample collected at each time point from this patient.

Changes in VEGF and PlGF over time were then assessed by univariate regression for each cancer type. Angiogenic biomarker values at time 0 were used to normalize values of subsequent time points for each patient. As shown in Figure 1, the only significant treatment effect identified was a reduction in PlGF levels over time in patients with metastatic sarcoma (*P* = .005). Among the patients with sarcoma, the most significant reduction in PlGF was observed in the patient with metastatic hemangiopericytoma, a sarcoma of vascular origin. This patient demonstrated a radiographic response by Choi criteria (40% reduction in the sum of tumor densities), which is often used to assess radiographic responses for this type of sarcoma [10]. The radiographic and biomarker changes for this patient are shown in Figure 2A and 2B. 

In addition, the patient with sarcoma who did not benefit from treatment demonstrated a significant reduction in PlGF levels over time (P = .006). This supports the hypothesis that cancer type predicts reduction in the PlGF biomarker independent of clinical benefit.

### *In vitro* sensitivity to Ang-(1–7)

The radiographic and biomarker findings highlighted hemangiopericytoma as being sensitive to treatment with Ang-(1–7). To study mechanisms of action, a murine endothelioma (EOMA) cell line was purchased. Although this model is not ideal, it is frequently used to study the biology of low grade vascular sarcomas [11-13].

**Figure 3:**
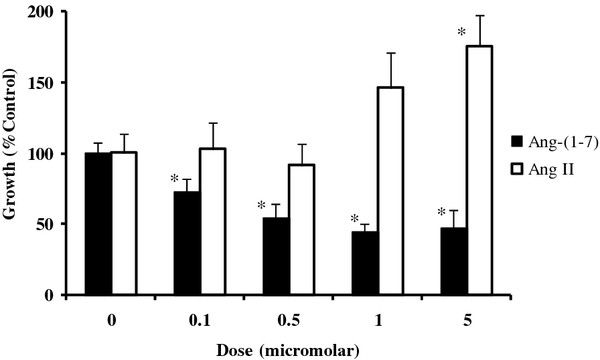
**Changes in the proliferation of murine endothelioma (EOMA) cells following treatment with either Ang-(1–7) (black bars) or Ang II (white bars) are shown.** * indicates a significant difference in proliferation (P < .05) compared to the proliferation rate of untreated cells.

As shown in Figure 3, treatment with clinically achievable doses of Ang-(1–7) reduced the proliferation of these cells at concentrations as low as 100 nM. The structurally similar eight amino acid peptide, Ang II, was applied to these cells at the same concentrations and did not reduce proliferation. At high doses, Ang II treatment stimulated proliferation of these cells. This indicates that the growth suppressive effects of the Ang-(1–7) peptide are due to a receptor mediated effect rather than a non-specific toxic effect of the peptide.

### Target gene analyses

To examine gene regulation responsible for the therapeutic effects of Ang-(1–7), murine PCR Arrays for angiogenesis and endothelial cell biology were performed. A total of 145 individual RNA species were measured by real-time PCR and compared in EOMA cells treated with Ang-(1–7) at a dose of 500 nM and untreated cells. Using a cut-off of a four-fold change in expression, 18 genes were identified as likely being regulated by Ang-(1–7).

**Figure 4:**
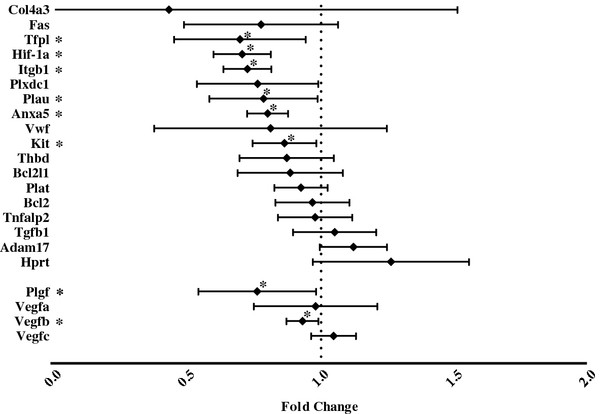
**Changes in expression of target genes following treatment with Ang-(1–7). Error bars represent the 95% confidence interval.** * indicates a significant difference in target gene expression (P < .05) in Ang-(1–7) treated cells as compared to untreated cells.

Confirmatory PCR Arrays were performed using at least four replicates for the purposes of statistical comparison. As shown in Figure 4, these analyses confirmed statistically significant changes in 8 genes including reduced expression of HIF-1α (*P* < .001) and PlGF (*P* = .04).

## Discussion

Phase I biomarker studies may fail to detect positive outcomes if a variety of cancers are treated and biomarker effects are dependent on cancer type. One approach to overcome this challenge is to perform multi-institutional phase I trials with eligibility restricted to a specific cancer type [14,15]. The approach taken for this study was to treat patients with a variety of cancer types and then test for the interaction of cancer type on biomarker outcomes. This approach has advantages as opposed to restricting eligibility when preclinical data have not established the most promising cancer type for clinical development.

Analyses of individual cancer types indicated that PlGF was reduced in patients with sarcoma and did not detect this effect in other cancer types. However, due to the very limited sample size within each group (n = 2 to 6), it cannot be concluded that biomarker changes do not occur in other cancer types. Despite the limited statistical power, identification of a significant biomarker effect in patients with sarcoma heightened our interest in the use of this drug for the treatment of this disease. Based on these biomarker findings and clinical outcomes, a phase II trial of Ang-(1–7) has been initiated to evaluate clinical activity and PlGF effects in patients with metastatic sarcomas.

The reverse translation of clinical findings back to a pre-clinical model is essential for ongoing development of this drug. The activity of Ang-(1–7) was tested in the cell line that most closely resembled the cancer type with the most substantial biomarker changes. This work revealed significant sensitivity at clinically achievable doses which provides additional rationale for clinical testing of this drug to treat hemangiopericytoma which is a very rare disease. In addition, this model has provided a platform for investigating a clinically relevant mechanism of action. This model uncovered HIF-1α as a probable mediator of the therapeutic effects in this disease [16-18]. This unique mechanism of action provides an attractive rationale for future combination of Ang-(1–7) with other anti-angiogenic drugs.

Angiotensin-(1–7) [Ang-(1–7)] and angiotensin II (Ang II) are structurally similar but bind distinct G-protein coupled receptors with opposing physiologic functions [3,19]. Ang II increases angiogenesis in animal models and human diseases while Ang-(1–7) reduces angiogenesis [3]. Recent studies have linked the pro-angiogenic effects of Ang II to upregulation of HIF-1α [20,21]. Taken together with our findings that Ang-(1–7) decreases expression of this gene, HIF-1α expression appears to be a key mediator of both the pro-angiogenic effects of Ang II and the anti-angiogenic effects of Ang-(1–7).

HIF-1α is the major censor of hypoxia that promotes angiogenesis by increasing expression of vascular endothelial growth factor (VEGF), placental growth factor (PlGF), and other pro-angiogenic factors [16-18,22-24]. Some of these pro-angiogenic hormones may also feedback to regulate HIF-1α expression and function [25]. It is likely that the reduction in plasma PlGF following treatment with Ang-(1–7) is triggered by the reduction of HIF-1α in tumors. Additional mechanistic studies are planned as part of future work to confirm that a causal relationship exists between these gene expression changes.

## Conclusions

Exposure to high concentrations of Ang-(1-7) reduces the production of pro-angiogenic peptides in transformed vascular cells by reducing the expression of HIF-1α.

## Competing interests

W. Jeffrey Petty has a potential financial interest in the development and use of the peptide studied in this project and his interest is being managed in accordance with Wake Forest University School of Medicine policies.

## Authors’ contributions

WJP, MA, and VAV conceived and designed the experiments. WJP and VAV performed the experiments. WJP, JL, VAV, and AAM analyzed the data. WJP, JL, and AAM wrote the paper: All authors read and approved the final manuscript.

## Pre-publication history

The pre-publication history for this paper can be accessed here:

http://www.biomedcentral.com/1471-2407/12/404/prepub
